# Editorial: Advances in plant diversity and its impact on high-nutrition and functional food

**DOI:** 10.3389/fpls.2023.1272727

**Published:** 2023-08-29

**Authors:** Adrián González, Lena Gálvez Ranilla, Claudia Fuentealba

**Affiliations:** ^1^ Biomedical Research and Innovation Center (CiiB), Universidad de los Andes, Las Condes, Chile; ^2^ Biopolymer Research and Engineering Lab (BiopREL), School of Nutrition and Dietetics, Faculty of Medicine, Universidad de los Andes, Las Condes, Chile; ^3^ Laboratory of Research in Food Science, Universidad Catolica de Santa Maria, Arequipa, Peru; ^4^ Escuela Profesional de Ingeniería de Industria Alimentaria, Facultad de Ciencias e Ingenierías Biológicas y Químicas, Universidad Catolica de Santa Maria, Arequipa, Peru; ^5^ Escuela de Agronomía, Facultad de Ciencias Agronómicas y de los Alimentos, Pontificia Universidad Católica de Valparaíso, Quillota, Chile

**Keywords:** agrodiversity, edible plants, bioactive compounds, phytochemicals, health

Awareness among consumers regarding the importance of incorporating nutritious and functional foods into their diets is growing (Wang and Zhang, 2023). Additionally, as the population increases, so does the demand for nutrients. Edible plants play a vital role in providing essential macro- and micronutrients, as well as bioactive compounds, which have physiological effects that contribute to disease prevention and improved human health.

Promoting plant biodiversity allows us to obtain a wide range of phytochemicals. However, it is becoming increasingly important to efficiently utilize plant resources to maintain a high production of nutrients and bioactive compounds under the current climate change context. This Research Topic covers various aspects related to the nutritional and functional potential of several plant-derived foods such as giant embryo rice, turmeric, basil, berries, and Native American plants. It contains four original research articles and one review.

In the first article, Hu et al. investigated the impact of nitrogen fertilizer on the yield and quality of giant embryo rice, and its effects on hyperlipidemia in an animal model (rats). Increasing nitrogen levels had a positive effect on rice yield, protein content, and gamma-aminobutyric acid (GABA) content. However, this effect was limited to a certain dose, as higher nitrogen levels led to reduced yield and could potentially cause environmental pollution. In addition, both of the studied giant embryo rice varieties decreased the body weight of rats and lowered the serum-related indices. These findings suggest that increasing nitrogen levels can be a simple and effective method for achieving high yield and GABA content in giant embryo rice, providing valuable insights for the promotion of giant embryo rice cultivation.

In the study conducted by Dudekula et al., the diversity among 200 Indian turmeric accessions was explored in terms of curcuminoid content and rhizome yield traits. Turmeric (*Curcuma longa* L.), a significant crop in Asia, particularly India, is known for its adaptability to different agroclimatic zones and its various beneficial properties such as anti-inflammatory, antioxidant, and antimicrobial effects. Additionally, turmeric holds cultural significance and is valued for its nutritional benefits. Correlation analysis revealed a significant positive relationship between the total curcuminoid content and both the primary rhizome core diameter and the length of the secondary rhizome. This suggests that these traits could be targeted for the improvement of turmeric breeding programs to develop new varieties with higher rhizome yield and increased curcuminoid content. Overall, the findings of this study highlight the genetic diversity present in Indian turmeric accessions, which can be utilized for future enhancement efforts to produce turmeric varieties with improved yield and curcuminoid content.

Tabbert et al. investigated the growth and chemical composition of four basil cultivars under different LED light intensities. The researchers focused on the development of volatile organic compounds and biomass efficiencies over time. The growth patterns of the basil cultivars varied depending on the cultivar type. A high light intensity condition accelerated the growth and increased the yield of all cultivars, making them more marketable. On the other hand, a low light intensity condition resulted in visually appealing qualities preferred by consumers, especially in green-leafed cultivars. These cultivars demonstrated better adaptation to low light conditions, leading to greater biomass efficiencies. Considering the superior visual quality of green-leafed cultivars and the significantly reduced energy consumption under the low light condition, basil producers may ultimately generate higher revenues.

The effect of different drying methods on the biological properties of Chilean murta shrub was evaluated by López et al. A freeze-drying process for berries exhibited higher total phenolic content and superior antioxidant potential when compared to other drying methods. On the other hand, vacuum-drying and infrared-drying were identified as viable alternatives, as they produced high-quality dried murta berries with the remarkable ability to preserve the anti-tumoral effect on cancer cells. Additionally, when it came to anti-inflammatory activity, freeze-drying and vacuum-drying proved to be the most effective. These findings demonstrate favorable drying methods for the production of dried berries with outstanding biological properties, highlighting the growing interest in improving the nutritional and functional quality of foods and increasing the diversity of edible plant cultivation.

The review by Winstead et al. provides a comprehensive analysis of Native American food plants (NAFPs) and emphasized the need to increase the attention from researchers and industry leaders on such valuable resources. The valorization of NAFPs not only holds potential for enhancing nutritional and ecological resilience but also for promoting the voice and culture of many Native American nations and conserving native biodiversity. This underscores the importance of recognizing and valuing the rich heritage and knowledge associated with NAFPs.

In conclusion, this Research Topic covers various aspects of plant diversity, including cultivation, nutrition, and functional foods. Four articles included in this topic focus on regions known for their high diversity of biological resources, such as China, North and South America, and India. This geographic diversity allows for a better understanding of the nutritional and overall quality potential of these regions, which can contribute to both local and global food security. Additionally, two research articles present innovative approaches to efficiently harness energy resources for food production in the face of climate change. The combination of exploring underutilized biological resources and implementing innovative technologies is crucial in addressing current challenges, including climate change, population growth, persistent malnutrition, and the increase of non-communicable diseases. Thus, these articles make significant contributions toward tackling these issues.

## Author contributions

AG: Conceptualization, Writing – original draft. LR: Conceptualization, Writing – review & editing. CF: Conceptualization, Writing – review & editing.

## References

[B1] Wang J. Zhang X. (2023). The big food view and human health from the prospect of bio-manufacturing and future food. Front. Nutr. 10. doi: 10.3389/fnut.2023.1160743 PMC10286865 37360295

